# Passive Mixing inside Microdroplets

**DOI:** 10.3390/mi9040160

**Published:** 2018-04-01

**Authors:** Chengmin Chen, Yingjie Zhao, Jianmei Wang, Pingan Zhu, Ye Tian, Min Xu, Liqiu Wang, Xiaowen Huang

**Affiliations:** 1 Energy Research Institute, Qilu University of Technology (Shandong Academy of Sciences), Jinan 250014, China; chencm@sderi.cn (C.C.); wangjm@sderi.cn (J.W.); zhupa@sderi.cn (P.Z.); xumin@sderi.cn (M.X.); lqwang@hku.hk (L.W.); 2 Key Laboratory of Biobased Polymer Materials, Shandong Provincial Education Department, School of Polymer Science and Engineering, Qingdao University of Science and Technology, Qingdao 266042, China; yz@qust.edu.cn; 3 Department of Mechanical Engineering, The University of Hong Kong, Hong Kong, China; tianye@hku.hk

**Keywords:** passive mixing, microdroplets, multiphase, simulation model

## Abstract

Droplet-based micromixers are essential units in many microfluidic devices for widespread applications, such as diagnostics and synthesis. The mixers can be either passive or active. When compared to active methods, the passive mixer is widely used because it does not require extra energy input apart from the pump drive. In recent years, several passive droplet-based mixers were developed, where mixing was characterized by both experiments and simulation. A unified physical understanding of both experimental processes and simulation models is beneficial for effectively developing new and efficient mixing techniques. This review covers the state-of-the-art passive droplet-based micromixers in microfluidics, which mainly focuses on three aspects: (1) Mixing parameters and analysis method; (2) Typical mixing element designs and the mixing characters in experiments; and, (3) Comprehensive introduction of numerical models used in microfluidic flow and diffusion.

## 1. Introduction

Microfluidics, working as a versatile, miniaturized, and integrated technology, has been widely used in many fields, such as chemical analysis, biological detection, drug screening, artificial photosynthesis, and microelectronics [1,2,3,4,5]. Mixing is necessary for these systems, particularly for those that involve heat transfer, mass transfer and chemical reaction. However, limited by the dynamic characters in microfluidics systems, most of the mixing efficiency is low and results in several problems. First, low mixing efficiency does not match the high-throughput requirements in quick analysis process, such as polymerase chain reaction (PCR) [6]. Second, low mixing efficiency may lead to heterogeneous mixture, causing low detection accuracy or poor product quality. Thus, the rapid homogeneous mixing is crucial in Lab-on-a-chip (LOC) platforms for widespread reactions covering biochemistry analysis, drug delivery, sequencing or synthesis of nucleic acids, cell activation, enzyme reactions, and protein folding.

**Figure 1:**
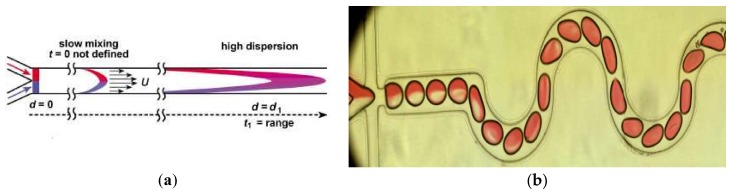

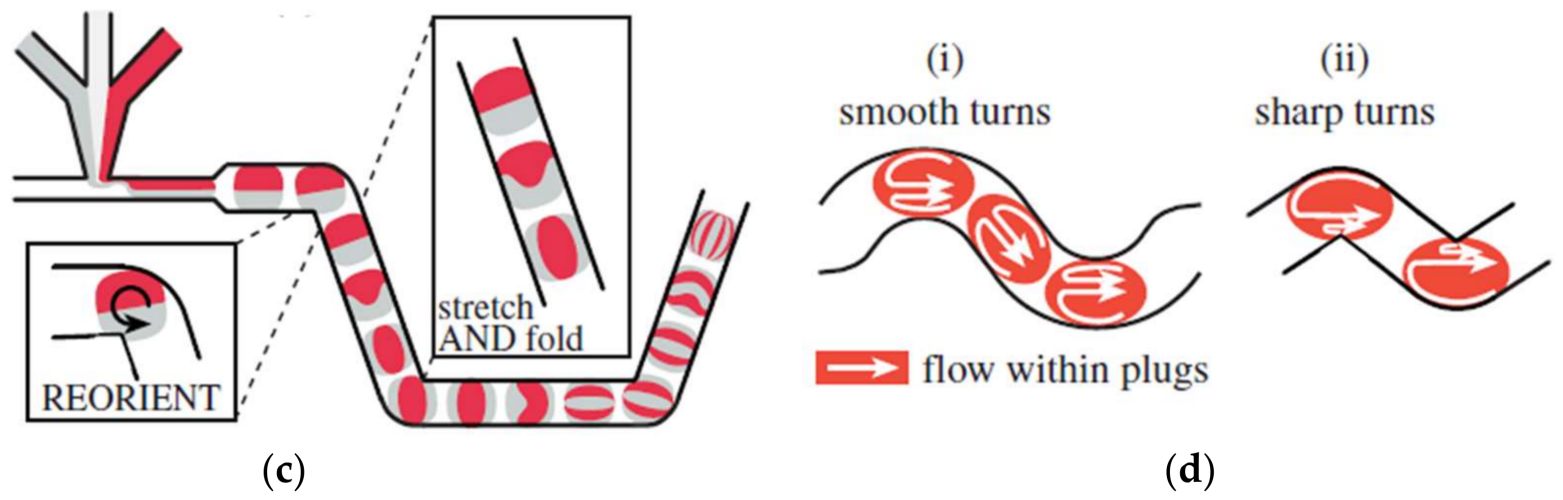
(**a**) Dispersion of solutes along the channel, reprinted with permission from [7]. (**b**) Microscopic image of the droplet in curved channel. (**c**,**d**) Recirculating flow scheme of Baker’s transformation in curved channel, reprinted with permission from [32].

According to the fluidic states in the mixers, mixing can be classified as single-phase mixing and droplet-based mixing [7]. In the single-phase mixing process, two or more reagents were injected into the microfluidic channels, and the mixing occurred by the diffusion between the fluid interfaces. Thus, the efficiency in single-phase mixing is limited by the diffusion flux, and dispersion of solutes along the channel is large (Figure 1a). Many methods have been introduced to overcome this limitation, such as via Split-and-Recombine [8], Chaotic flow [9], and nozzles [10]. When compared with single phase mixing, droplet-based mixing was put forward to overcome the drawbacks of low mixing efficiency in single-phase and high solutes dispersion because of internal recirculation and isolated environments [11,12]. In the droplets-based mixers, two or more kinds of reagents are driven into the channel independently and meet in the junction. The two immiscible phases used for the droplet generation are referred to as the continuous phase (medium in which droplets are generated) and dispersed phase (the droplet phase). With the cooperation of the geometry of the junction, the flow rates and the physical properties of the fluids the local flow field is determined, which leads to the interface deformation and droplet formation [13]. After the droplet has formed, the mixing begins. Because of its special performance when compared with the single phase mixing, droplet-based mixing plays a worthy role in the processes of chemical reactions [14,15,16], biological synthesis, and diagnostics [17,18,19,20], especially for miniaturized reactions or diagnostics [21]. 

**Figure 2:**
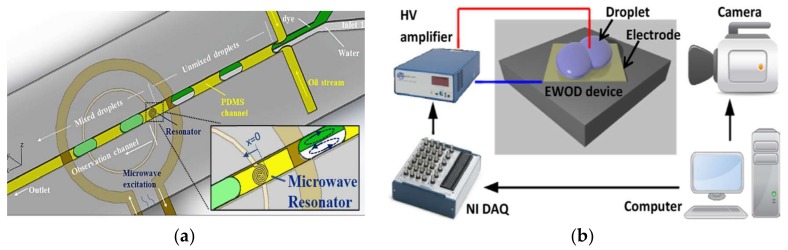
Droplet mixing in a microfluidic device (**a**) Mixing enhanced by adding a microwave heater: the heat induces non-uniform Marangoni stresses, leading to fast mixing, reprinted with permission from [25]. (**b**) Droplet mixing by alternating current (AC) electrowetting: the best mixing time was about 2% of the diffusive mixing time, reprinted with permission from [26].

However, it is also difficult to achieve a good mixing performance for droplets-based mixing in a straight channel, where the flow is laminar flow (*Re* = 0.01~100) [22]. The principle of intensifying the mixing is the increase of the diffusive flux between different disperse phase reagents, which is affected by the diffusion coefficient, interfacial surface area, and concentration gradient [23]. There are several devices designed to enhance the mixing in droplets, and they can be classified into two categories: active mixers and passive mixers. (1) In the active mixers, some external physical field is introduced to improve the efficiency via the external energy-induced eddy diffusion and bulk diffusion in the droplets [24]. Mixing in this mode relies on the materials’ (those inside the fluid) response to the external physical field. Yesiloz et al. presented a microwave-based mixer, which heats the droplet by microwaves and induces non-uniform Marangoni stresses. In this mixer, highly viscous fluid (75% (*w*/*w*) glycerol solution) was selected as dispersed phase. By seeding with a fluorescent in half of the droplets, the mixing performance was investigated and the results showed that the mixing index reached as high as 97% within milliseconds [25] (Figure 2a). Bansal, et al. did some researches on the droplet mixing, depending on the non-axisymmetric oscillation patterns induced by actuation parameters in AC electrowetting, and the results show that the best mixing time in this system was approximately 2% of the diffusive mixing time [26] (Figure 2b). Besides these external energy sources, others are also used, such as magnetic field [27,28] or acoustics [29]. (2) In passive mixers, the mixing is achieved by the droplet movement only, and none of these structures employ external energy apart from the pump drive [30,31]. A simple scenario is making the droplets large enough to overfill the channel and exhibit a pancake shape, thus the mixing is intensified due to the shear forces between the wall and droplet interface. However, in this condition, the recirculation is enhanced only at one disperse phase side, and the mixing between two disperse phase reagents is limited. Therefore, some researchers introduced “Chaotic flow” into the droplet mixing process to break the limitation of the laminar regime. The most commonly used scheme is the channel deformation to realize the “Baker’s transformation” in the droplets (Figure 1b–d) [32]. Figure 1b is the mixing process in one of curved channels. In these channel, the droplet stretched and folded inside the turns (sketched in Figure 1c), leading to asymmetrical recirculation in the droplets (shown in Figure 1d). In the deformation channels, the “Chaotic flow” in droplets is enhanced by stretching, folding, and rotation. 

Active mixers and passive mixers have their own advantages, respectively. For instance, active mixer presents their advantages in precise control and high efficiency, as well as high viscosity liquid mixing both in enclosed channel and on the substrate surface. They are also applicable for highly viscous fluid. Passive mixers show their advantages in terms of (1) reliable manipulation: the passive mixing relies on droplet movement in the immobile channel without any external energy and the external energy-induced instability [23]; (2) moderate reaction condition: external energy (e.g., heat or electric field) in the droplets may destroy some fragile molecules or deactivate some sensitive biomolecules; and, (3) easy fabrication of devices. With these merits, passive mixers are already applied in DNA hybridization analysis, polymerase chain reaction (PCR), cell activation, and chemical analysis [12,16,21] A brief comparison is showed in Table 1.

In the following sections, we will briefly review the microdroplet-based passive mixer, including three parts: (1) Mixing parameters and analysis method, (2) Typical designs and mixing characters in experiments, and (3) Comprehensive introduction of numerical models that are used in the microfluidic flow and diffusion.

## 2. Characterization of Mixing in Microdroplets

Two parameters are important to characterize the mixing performance: the mixing time/mixing length and the distribution uniformity. When compared with the mixing time/mixing length, the distribution uniformity is more difficult to evaluate.

The most common and simplest way to measure the mixing uniformity is visible imaging, via flow visualization experiments with the aid of photometric, fluorescence intensity measurements [33,34], particle image velocimetry (PIV) measurements [35,36,37], high-speed color imaging [26], laser-induced fluorescence (LIF) [38,39], spontaneous Raman scattering (SRS), and many others [30]. However, the reliability of techniques and instruments limit the accuracy of visualization. Theoretical evaluation is a good solution for this problem. Most of evaluation methods are based on the principle of the non-homogeneous level of the tracer concentration distribution in a certain droplet. Standard deviation is an important parameter reflecting the uniformity of the tracer distribution, and sometimes it is adopted directly as a criterion of the mixing efficiency (Equation (1)) [40]. In Equation (1), $\sigma_{c}$ is called standard deviation. When *N* is replaced by *N* − 1 [41,42], $\sigma_{s}$ is called sample standard deviation, which is closer to the real deviation value in analyzing samples. The *C* is Concentration value, *C_d_* is the reference concentration value.

(1) $$\sigma_{c}=\sqrt{\frac{1}{N}\sum_{i=1}^{N}{\left(C-C_{d}\right)}^{2}}\;\mathrm{or}\;\sigma_{s}=\sqrt{\frac{1}{N-1}\sum_{i=1}^{N-1}{\left(C-C_{d}\right)}^{2}}$$

However, it is hard to compare different conditions using the standard deviation. During the last few years, several studies have been conducted on different micromixers that are aiming to characterize mixer performance. The widely used definition is the Danckwerts’ segregation intensity index based on the variance, referred to as mixing index or mixing efficiency (m), as expressed by Equation (2) [43].

(2) $$m=1-\frac{\sigma_{c}}{\sigma_{c,max}}$$

The concentration profiles are discrete functions. Therefore, Equation (2) is replaced by Equation (3) in experimental measurements [44,45],

(3) $$m=1-\frac{\sqrt{\frac{1}{N}\sum_{i=1}^{N}{\left(C-C_{d}\right)}^{2}}}{C_{d}}$$

Other dimensionless numbers are also used based on the variance or standard deviation, such as intensity of segregation (IOS) [46] values and χ [34,47]. The symbol definition refers to the symbol list.

(4) $$\mathrm{IOS}=\frac{\sigma_{C\prime }^{2}}{C_{d}^{\prime }\left(1-C_{d}^{\prime }\right)}$$

(5) $$\chi =\frac{\sigma_{c,t}-\sigma_{ref}}{\sigma_{c,0}-\sigma_{ref}}$$

In addition to these quantification methods, there are other novel characterization techniques. In the reaction systems, the reaction processes is accompanied by the enthalpy and kinetics change [48], so the dissipated energy is used to estimate the intensive mixing time. If the reaction is included, other indexes, such as pH value, are also used to evaluate the mixing performance according to the reaction type [49,50,51].

In simulations, mass fraction is usually used to estimate the mixing uniformity, since very detailed information can be obtained [44,52]. Similar to the experimental method, the same evaluation method is used. Because the mass fraction is a relative value in a certain area, the integral form is used in the equations (Equations (6) and (7)) [53,54]. In addition to the mass fraction, the particle tracer method that is based on the particle distribution in the droplet is usually used [55,56]. In this method, the particle location is varied with the mixing progress, and the uniformity of the particle location may represent the mixing uniformity to some extent.

(6) $$m=\left(1-\sqrt{\frac{\iint {\left(C_{f}-C_{f,d}\right)}^{2}dA}{A\cdot C_{d}\left(C_{f,max}-C_{f,d}\right)}}\right)\cdot 100\%$$

(7) $$m=\left(1-\frac{\iint |C_{f}-C_{f,d}|dx\mathrm{dy}}{\iint |C_{f,0}-C_{f,d}|dx\mathrm{dy}}\right)\cdot 100\%$$

## 3. Micro-Mixers Design and Experiment Study

Passive mixing, without extra energy, relies on the molecular diffusion and chaotic advection in the droplet [57]. In the straight channels with two symmetrical streams, the formed droplets contain two symmetrical circulating flows on each half of a droplet, so it is difficult to mix together. Varying the channel geometries is the most effective strategy to enhance the mixing [57], and two methods were therefore proposed. One is to change the way of droplet formation, and the other is to change the way of droplet movement along the channel [12].

### 3.1. Mixing during Droplet Formation

To improve the mixing efficient, one can take full advantage of the internal recirculation inside the droplet during its generation. 

**Figure 3:**
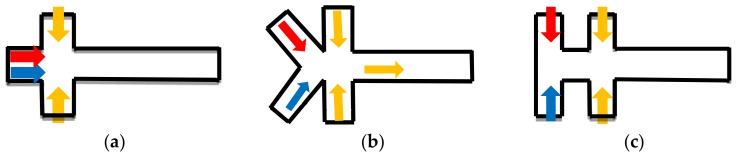

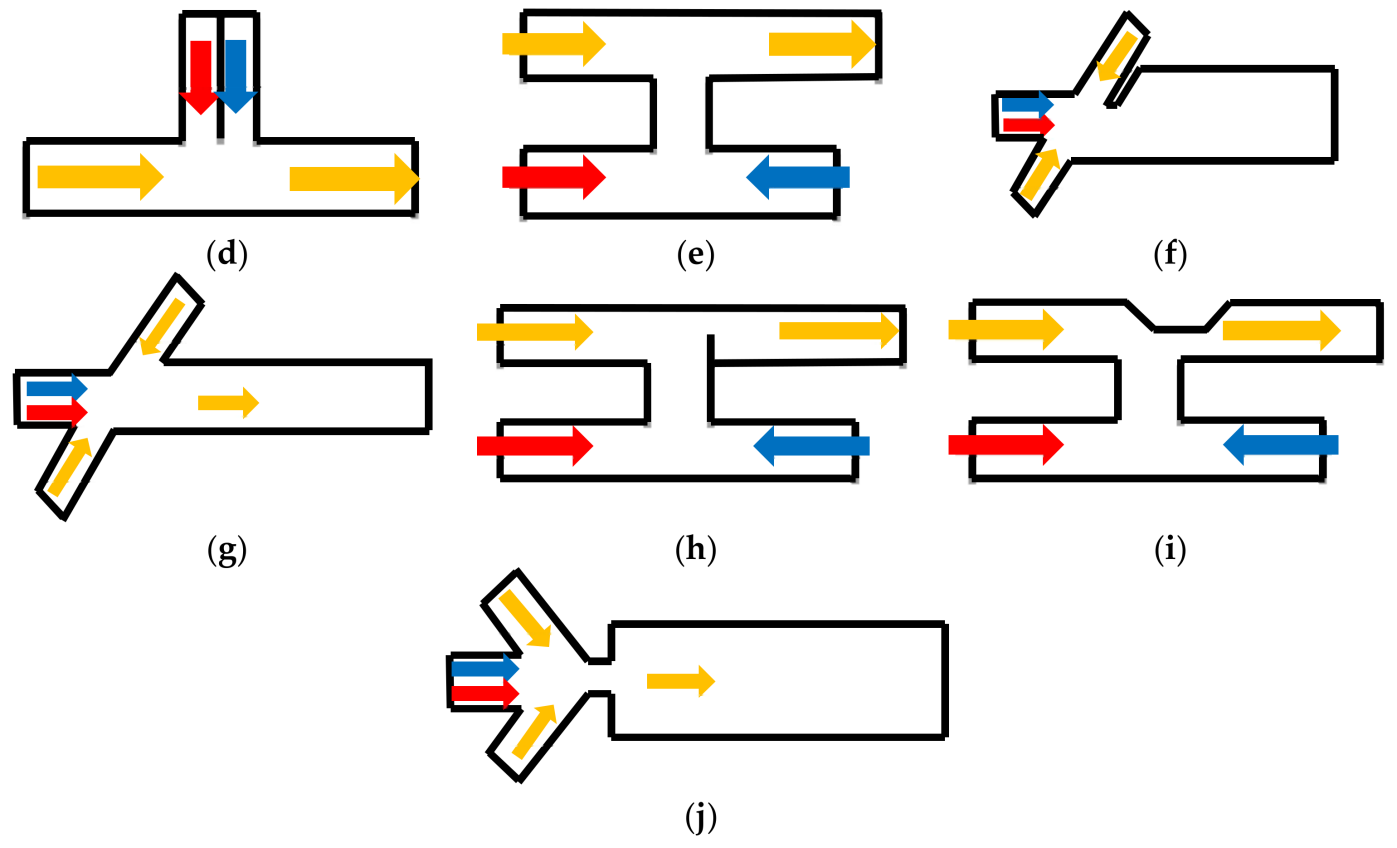
The schematic figures of the different generation sections. (**a**–**c**), three types of the asymmetric inlets model. (**d**–**i**), six types of the “convergent–divergent” model [61].

For droplet mixing in the microfluidic devices, at least two dispersed phase inlets and one continuous phase inlet are required. The spatial location of the dispersed-phase inlets affects the fluid distribution inside the droplets, which may have a strong influence on the mixing performance [58]. The droplets that are formed by a bilateral symmetric fluid distribution structure take a long time to mix due to the low speed of diffusion between the two phases (Figure 3a), and some modification was proposed to improve the mixing in the cross junction (Figure 3b,c). Although the dispersion phase distribution is symmetrical in the droplets formed by these type, the droplets are more sensitive and the symmetry is more vulnerable to breaking, which leads to high mixing efficiency. Lin Bai studied the mixing efficiency of “Y-junction” in the mixing of ionic liquid (IL) droplets, and it is about 0.75 when compared with 0–0.4 in cross junction [12]. Wang, et al. studied the cross-shaped microchannel in Figure 3c. It is found that the IOS of the droplets generated in this channel has a low initial value [57]. Asymmetric dispersed phase inlets distribution [46,59,60], for example, “T-junction” (Figure 3d,e), is one of the possible methods to break the bilateral symmetric fluid distribution. Lin Bai also compared the mixing efficiency of “T-junction” with that of the cross section in the same conditions. It is verified that mixing efficiency is about 0.4–0.7, which is higher than 0–0.4 in cross junction. However, in this type, the reagent contacts the wall in the droplet formation process, which may have negative effects on the samples [54], so another asymmetric type was designed to overcome this drawback (Figure 3f,g). In these structures (Figure 3f,g), the advantage in enhancing the mixing performance is that the asymmetric design caused the vortex flows in the droplet formation, which leads to 1.5 times higher mixing index when compared with conventional flow-focusing structures [54].

Another improved structure in the droplet formation section is to design a sudden shrink and enlarge the channel near the droplets formation location (Figure 3h–j). In “convergent–divergent” channels, the local flow speed is increased and the formation of the swirling structure in droplets is also sped up [12,54,62]. 

### 3.2. Mixing during Droplet Transportation

In the mixing process, fluid parameters and the microchannel structures impact the mixing performance obviously. Song, et al. [32] proposed a scaling law for the dependence of the mixing time *t*~(*aw*/*V*)ln(*Pe*) by the experiment of droplets mixing in meandering channels, where *a* is the dimensionless length of the plug measured relative to *w*. The discussion was useful to choose the proper operation condition. In a certain microchannel structure, *a* is important to the mixing performance. A smaller size (*a* < 1) results in a shorter mixing time/distance because of the high circulation speed. For the droplet that is large enough to contact with the channel walls, the mixing relies on the recirculation that is caused by the wall-induced shear stress. Wang, et al. [53] shows that when 1 < *a* < 2, the asymmetric circulations make the disperse phase easy to mix in droplets moving in meandering channels. However, when *a* > 2, the asymmetric circulations had little effect on enhancing the mixing efficiency, which corresponds to the experimental results from Harshe, et al. [45]. In the following section, we focus on the mixing in the droplets with a diameter that is comparable to the channel width.

**Figure 4:**
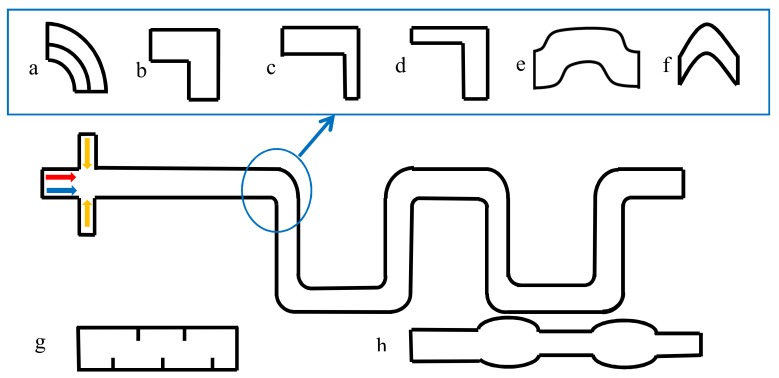
Deforming microchannels, (**a**–f) different structures of the meandering microchannel, (**g**) obstacle arrangements inside a microchannel, (**h**) the structure of a deforming channel.

Microchannel structures deformation was widely used to break the symmetric recirculation in the droplets moving in the straight channel. The common methods are meandering channels (Figure 4a–f) or obstacle arrangements (Figure 4g) to break the symmetrical recirculation as well as to increase the effect of chaotic advection for mixing [34,53,56,58,63,64,65].

The dispersed phase reagent is reorientated within each turn of the curved channel. With the help of the reorientation, the mixing performance is enhanced. The obstacles function in the same way, which causes the asymmetrical circulation in the droplets. In some structures, the curved channel is combined with the “convergent–divergent” channel to enhance the mixing, and the results are good (Figure 4c,d). Tung et al. [66], did some experimental research about the droplets mixing in these structures in Figure 4c–e with the help of the high-tempo micro-particle image velocimetry (l-PIV). The results show that the mixing index increased eight times when compared with the straight microchannel at the same Reynolds number (Re = 2). The angle design of the turn is also very important for the enhancement of mixing efficiency (Figure 4e,f). Sarrazin, et al. [34] estimated the mixing performance in droplets using *χ* (Equation 5), and the results show that the channels with angles of 45° and 90° show good mixing efficiency (mixed within 10 ms) when compared with the straight channel or with an angle of 135° (mixing time is about 70 ms). Jiang, et al. [43] studied mixing efficiency in droplets moving in the channel with an angle of 60°, and the mixing time is about 18 ms when efficiency reached 80%. Besides meandering channels, other deforming channels were also presented (Figure 4g,h). In Figure 4g, the baffles were installed in the channel to change the position of the two independent circulation areas inside the large droplets [56], with the modification, the mixing efficiency decreased little with the droplet size increased. In Figure 4h, the droplet mixing is enhanced with the complex vortices generated by the droplet deformation when it crosses the compressed and expand areas alternatively [12].

**Figure 5:**
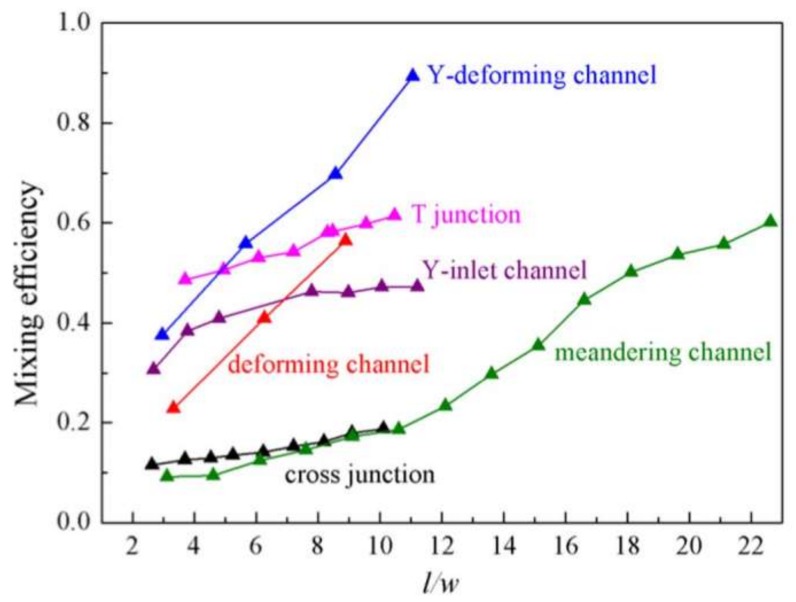
Mixing efficiency comparison in different micro-channels, reprinted with permission from [67].

Bai, et al. [12] gave detailed cooperation about the mixing performance inside IL droplets moving in various microchannels under the same flow conditions (Figure 5). Such a comparison demonstrates that although mixing inside IL (with viscosity of 66.4 mPa⋅s (25 °C)) is much more difficult than that in regular fluids, the design of combining the Y inlet (Figure 3b) and deforming channel (Figure 4h) could enhance the IL droplet mixing efficiency to high values that is close or even better than regular fluid droplets in common channels.

The most important principle to enhance the mixing performance is to break the symmetrical distribution of the disperse phase in the droplets. “Y” type and “T” type are the most used inlet structures with good mixing efficiency. In the meandering channels, small turn angles have good mixing performance. Good mixing performance can be achieved by combining the improved inlet structure and channel design. 

## 4. Numerical Simulation

Computational fluid dynamics (CFD) is widely used to investigate the transport process in droplet-based mixing [68] for a comprehensive understanding of this process. In the droplet mixing processes, the multiphase model and the species transport model are used for investigation. 

Droplet formation has been studied by a few researchers using the conventional CFD methods [68], including Volume of fluid method (VOF), Level Set method (LSM), Lattice Boltzmann method (LBM), and so on [54], while the species transport model is commonly used in the diffusion process. The most used multiphase flow models and species transport models are listed in Table 2.

### 4.1. Volume-of-Fluid (VOF) Model

**Figure 6:**
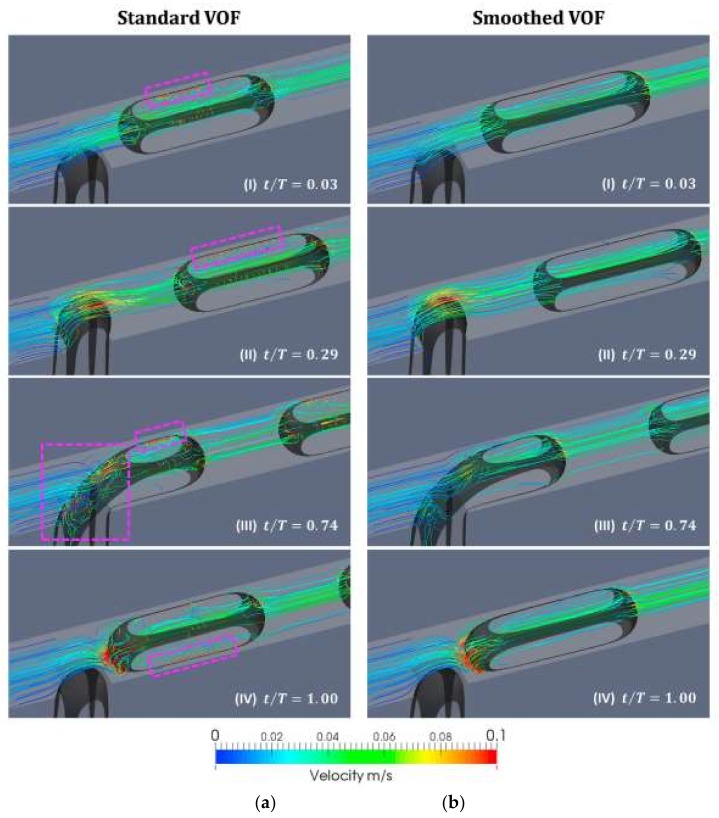
The flow fields near the interface during the droplet formation process, by (**a**) the Volume of fluid method (VOF) method and (**b**) the improved VOF method. Spurious velocities appeared near the interface in the VOF model. Reprinted from [67].

VOF [77] model is based on the fact that different phases are not interpenetrating, meaning that the fraction of the fluid volumes are addable in computational cells. It solves the surface flow with minimum consumption of computational resource, because this model solves a single set of Navier–Stokes differential equations for all of the phases and relies on the reconstruction of the interface by solving an advection equation (Table 1) [78]. However, because the VOF model suffers from the spurious velocities that are introduced from the computation of the mean curvature (Figure 6), its accuracy is poor in tracking the information of the interface [79]. Couplings with the improved interface tracking algorithm improve the VOF model accuracy [67,80]. 

### 4.2. Level Set Method (LSM)

Level set method is an iterative, numerical technique to capture the interfaces and shapes within a fixed grid system [81]. In the level set method, an interface is represented by a contour of a smooth scalar field where ϕ = 0, ϕ > 0 and ϕ < 0 represent two different phases.

**Figure 7:**
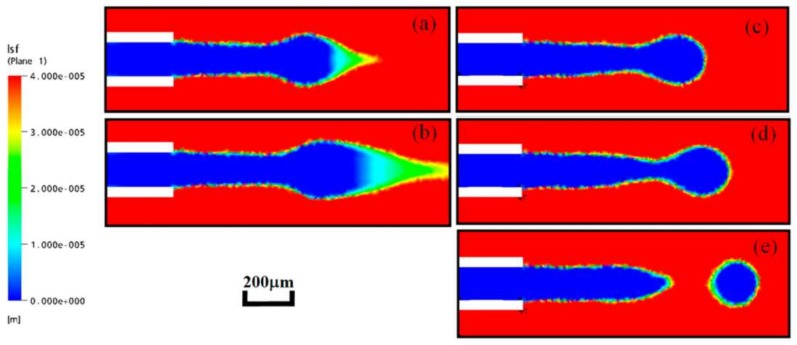
Simulation results comparison between the improved level set method and original level set method: (**a,b**), simulated by original level set method, (**c**–**e**), simulated by improved level set method. Reprinted with permission from [82].

When compared with the VOF model, the advantage of the level set method is the complex topological changes and computing with surface tension [82]. However, the level set function may be distorted by the flow field after some iteration steps, which leads to inaccurately approximated values on the interface (Figure 7). As a result, the simulation results may go against the mass conservation. To overcome this difficulty, some improved methods of the level set method is developed, such as dual-resolution LSM, sharp-interface LSM and conservative LSM [82,83].

### 4.3. VOF Coupled with Level Set Method (CLSVOF)

**Figure 8:**
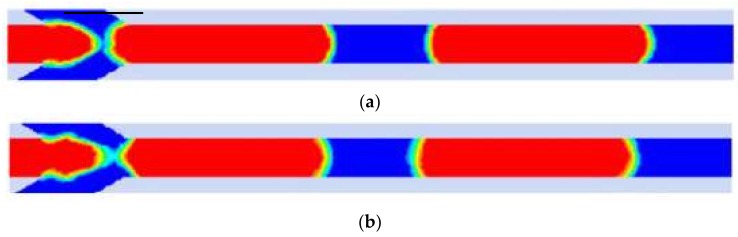
Comparison between (**a**) VOF Coupled with Level Set Method (CLSVOF) method (**b**) and VOF method. The interface in (**a**) is smoother than that in (**b**). Reprinted with permission from [73].

CLSVOF is a hybrid method combining both the level set method and the volume-of-fluid model [84]. This cooperation gets an improvement of the mass conservation as well as a more accurate interface-tracking (Figure 8). However, the hybrid method has a risk in numerical instability at the interface region when the interfacial tension is a dominant factor in complex geometries.

### 4.4. Lattice Boltzmann Method (LBM)

LBM, based on the Lattice Boltzmann equation, is developed from the discretized fluid model Lattice Gas Automata (LGA) [56,85,86]. The nature of the LB model is based on the assumption that molecular clusters are restricted at the discrete set of lattices and the molecular clusters act at each lattice side in two steps: collision and streaming [87]. LBM is positioned between the continuum level (described by Navier–Stokes equations) and the microscopic (molecular) level. The LBM could be classified to several types [88], such as color-fluid model [87,89], the pseudo-potential model [90], and the free-energy model [91]. LBM can successfully capture the motion and deformation of the interface and has been applied to simulate two-phase flows in microchannels. But, there are some drawbacks in these methods, such as the complexity and time-consumption in the coloring and recoloring step; the pseudo-potential model works well for low-density ratios only; and, the free-energy approach has a lack of Galilean invariance. That is why there are still other newly developed methods that are used in the droplet formation and mixing processes, according to different cases [92,93].

With the improvement of the simulation methods, the simulation results become more accurate, which plays an important role in the study of droplet mixing. The previous work has shown that, when compared with experiment results, each method can capture the droplets formation dynamics and the internal velocity fields inside the droplets successfully. When combined with species transport method, the simulation error in mixing efficiency is also acceptable. Yang, et al. compared the experiment and simulation results with LSM method, and in same condition, when mixing efficiency is 0.9. The mixing time of experiment and simulation is about 0.025 s and 0.027 s, respectively [63]. Jiang, et al. also did the comparison using LSM mothed, and the result is that when the mixing efficiency is 0.8, the mixing times of experiment and simulation are about 0.014 s and 0.018 s, respectively [43]. Wang, et al. studied the IOS of droplets mixing in meandering channels, the simulation results have good concordance with experimental results in the range of 0 < l/*w* < 10, even in some place, the IOS value coincided very well [58]. 

When we do the simulation works, we should choose the best models according to the simulation conditions, such as the accuracy of results, the flow situation, and the purpose. With the help of the simulation, the modifications of the structures and parameter studies are time- and cost-saving. Table 3 lists some numerical studies on mixing in recent years.

## 5. Conclusions

When compared with the active mixers, passive mixers have the advantages of lower cost, simple device design, and reduced power input, which make the passive mixers widely applicable. In this paper, we gave a brief review of the experimental and simulation results of the passive mixers based on droplets. Also, we systematically analyzed the quantification methods, newly developed types of mixers and the simulation methods. With the improved experimental devices and simulation methods, more detailed mixing principles and structure designs have been present. The results presented in this review will shed light on further study of droplet based-passive mixers. In the future, the innovative mixing technology will be more widely used integrated with other steps from sample-in to result-out. Besides that, there is still need for some studies on mixing for special applications, such as high viscosity, high heat release/absorb from reaction in the droplet, and so on.

## Figures and Tables

**Table 1:** Comparison of active and passive Mixers.

Categories	Principle	Mixing Time	Devices Fabrication	Stability of Operation	Application Scope
Active mixers	Disturbance caused by the external energy	Milliseconds	Complex, energy input including flow driven energy and mixing energy	Lower	The flow of response material or with response material
Passive mixers	Droplet movement in the immobile channel	Tens of milliseconds	Simple, only needs flow driven energy	Higher	All flow

**Table 2:** The difference of simulation models.

Method	Equations	Note	References
VOF and its improved methods	$\{\begin{array}{c} \nabla \cdot V=0\\ \frac{\partial \rho V}{\partial t}+\nabla \left(\rho V\cdot V\right)=-\nabla P+\rho g+\nabla \cdot \mu \left(\nabla V+\nabla V^{T}\right)+F\\ \frac{\partial \alpha }{\partial t}+\nabla \left(\alpha V\right)=0 \end{array}$ $\alpha \{\begin{array}{c} 0,\;empty\;of\;A\;phase\;\\ 0\sim 1,\;contain\;the\;interface\;\\ 1,filled\;of\;A\;phase \end{array}$	Interface representation 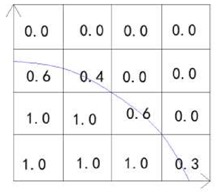	[7,60,69,70,71,72,73]
LSE	$\{\begin{array}{c} \nabla \cdot V=0\\ \frac{\partial \rho V}{\partial t}+\nabla \left(\rho V\cdot V\right)=-\nabla P+\rho g+\nabla \cdot \mu \left(\nabla V+\nabla V^{T}\right)+F\\ \frac{\partial \varphi }{\partial t}+V\nabla \varphi =0 \end{array}$ $\varphi \left(X,t\right)\{\begin{array}{l} d,\;if\;x\;in\;the\;liquid\;A\;phase\\ 0,\;if\;x\;in\;the\;interface\;\\ -d,if\;x\;in\;the\;liquid\;B\;phase \end{array}$	Interface representation Interface φ=0 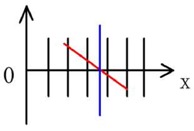	[55,63]
LBM	$f_{i}^{k}\left(x+e_{i}\delta t,t+\delta t\right)=f_{i}^{k}\left(x,t\right)+\Omega_{i}^{k}+G_{i}^{k}$		[74,75]
Specie transport	$\frac{\partial C_{i}}{\partial t}+V\cdot \nabla C_{i}=D\nabla^{2}C_{i}$		[76]

**Table 3:** Some studies on simulation of mixing performance in recent years.

Mixing Mechanism	Conditions	Evaluate Method	Mixing Performance	Model	Reference
Baffle in channel	*Pe* ~ 102; *Ca* = 0.0008~0.08; *Re* = 0.2~20	Standard deviation	When l/*w* = 6, $\sigma_{c}$ is about 0	LBM	[56]
Asymmetric inlets	*Re* = 0.39~2.93	Mixing index	When *t* = 14 ms, 0.90	VOF	[59]
	*Ca* = 0.06~0.006; *Re* = 0.1~0.001	Mixing index	When Ca = 0.06, 0.90	COMSOL Multiphysics	[54]
Serpentine microchannel	*V* = 1.11 m^3^/s; 2.22 m^3^/s; *D* = 100 μm/120 μm	Particle trajectories/time	0.08s	LSM	[55]
	*V*(A) = 0.005~0.04 m/s; *V*(B) = 0.01 m/s	Mixing index	*L*/*w* = 16, 0.90	LSM	[63]
	*D* = 50 mm; *Re* = 3.10; *Ca* = 0.0036	Mixing index	When *L*/*w* = 16~32, 0.90	VOF	[53]
Converging shape	*Ca* ~ 0.02; *V*(A) = 100 μL·min^−1^; *V*(B) = 10 μL·min^−1^	IOS	When *L*/*w* = 10 0.2~0.4	LBM	[46]
	*Ca* ~0.022; *Re*~2.5	IOS	When *L*/*w* = 10, 0.5	LBM	[58]

Note: A: continuous phase; B: dispersed phase.

## Abbreviation

Symbol	
*m*	Mixing index
*N*	The number of the samples
*C*	Concentration value
*A*	Area
*l*	Length of channel
*w*	width of channel
*D*	Channel diameters
$\sigma_{c}$	Standard deviation of concentration
$\sigma_{s}$	Sample standard deviation
*C_d_*	The reference concentration value
*t*	Time, s
$C^{\prime }$	Normalized concentration
$C_{d}^{\prime }$	The statistical average value of normalized concentration in the entire droplet
*C_f_*	Mass fraction
*C_f,d_*	The reference Mass fraction
*ρ*	Density
μ	Viscosity
*g*	Gravitational constant
*V*	Velocity
***V***	Vector of velocity
*Pe*	Peclet number
*C_a_*	Capillary number
*e*	Vector of lattice direction
*Re*	Reynolds number
*F*	Interaction force
*P*	pressure
$\Omega_{i}^{k}$	Collision operator
$G_{i}^{k}$	External force term
*α*	Volume fraction in VOF model
Subscrips	
0	The initial condition
max	The maximum value
t	The condition of certain time
∞	The condition in well mixing section
